# Revealing Novel-Strain-Specific and Shared Epitopes of Infectious Bronchitis Virus Spike Glycoprotein Using Chemical Linkage of Peptides onto Scaffolds Precision Epitope Mapping

**DOI:** 10.3390/v15112279

**Published:** 2023-11-20

**Authors:** Samantha Sives, Sarah Keep, Erica Bickerton, Lonneke Vervelde

**Affiliations:** 1 Division of Immunology, The Roslin Institute & R(D)SVS, University of Edinburgh, Easter Bush, Edinburgh EH25 9RG, UK; lonneke.vervelde@roslin.ed.ac.uk; 2 The Pirbright Institute, Ash Road, Woking GU24 0NF, UKerica.bickerton@pirbright.ac.uk (E.B.)

**Keywords:** coronavirus, epitope, infectious bronchitis virus, chicken, antibody response, spike

## Abstract

The avian coronavirus, infectious bronchitis virus (IBV), is an economically important infectious disease affecting chickens, with a diverse range of serotypes found globally. The major surface protein, spike (S), has high diversity between serotypes, and amino acid differences in the S1 sub-unit are thought to be responsible for poor cross-protection afforded by vaccination. Here, we attempt to address this, by using epitope mapping technology to identify shared and serotype-specific immunogenic epitopes of the S glycoprotein of three major circulating strains of IBV, M41, QX, and 4/91, via CLIPS peptide arrays based on peptides from the S1 sub-units. The arrays were screened with sera from chickens immunised with recombinant IBV, based on Beau-R backbone expressing heterologous S, generated in two independent vaccination/challenge trials. The screening of sera from rIBV vaccination experiments led to the identification of 52 immunogenic epitopes on the S1 of M41, QX, and 4/91. The epitopes were assigned into six overlapping epitope binding regions. Based on accessibility and location in the hypervariable regions of S, three sequences, ^25^YVYYYQSAFRPPNGWHLQGGAYAVVNSTN^54^, ^67^TVGVIKDVYNQSVASI^82^, and ^83^AMTVPPAGMSWSVS^96^, were selected for further investigation, and synthetic peptide mimics were recognised by polyclonal sera. These epitopes may have the potential to contribute towards a broader cross-protective IBV vaccine.

## 1. Introduction

The avian coronavirus, infectious bronchitis virus (IBV), is an endemic poultry pathogen, causing a highly contagious respiratory disease [1]. Economic losses associated with IBV are linked to secondary opportunistic bacterial infections [2,3,4]. As there are many different strains or serotypes of IBV circulating worldwide and with the continual emergence of new strains every few years [5], available vaccines are often not protective against the newly emerged variant strains. Consequently, it is common practice to use combinations of live or inactivated vaccines, from different serotypes of the virus, to improve the breadth and potency of cross-protection afforded against circulating field strains of IBV [6,7,8,9,10,11]. The order in which the different vaccines are given can also influence the level of cross-protection induced in flocks [12].

The issue of limited cross-protection between IBV serotypes is often associated with the variance in the major surface protein, spike (S), a type 1 glycoprotein which oligomerises to form trimers [13,14,15]. The S protein is proteolytically cleaved into two subunits, the N-terminal subunit S1 (approximately 500–550 amino acids, 90 kDa) and the C-terminal subunit S2 (approximately 610–630 amino acids, 84 kDa), which contains the transmembrane domain. The S1 subunit is solely responsible for binding to host cellular receptors [16,17], determines the serotype, and is responsible for the induction of neutralising antibodies [18,19,20,21,22], with the S2 conformation postulated to have an important role in the conformation of S1 [20,23,24]. The variance within the S protein, in particular the S1 subunit, is the major reason behind the emergence of new variants/serotypes and the continual issues of poor cross-protection between serotypes [25,26]. The S protein and S1 subunit can trigger a protective immune response in chickens vaccinated with recombinant S or S1 expressed in viral vectors, including adenovirus and recombinant IBV, evident by partial protection against homologous and heterologous wild-type viral challenges [20,27,28,29,30,31]. The role of the S2 subunit in the induction of protective immunity still remains to be elucidated, with vaccination studies reporting that S2 alone is unable to induce sufficient protection against a homologous challenge [20,32] and a heterologous challenge [33]. Some combinations of commercially available vaccines can induce cross-protection against unrelated serotypes, indicating a synergistic effect of some of the vaccines, which are referred to as protectotypes [34]. Collectively, this suggests that one, but more likely several, critical epitopes are responsible for the cross-protection conferred between some IBV serotypes. The identification of such epitopes is of paramount importance for the rational development of vaccines with the ability to induce broadly neutralising antibodies.

Several studies have identified neutralising epitopes present on the S glycoprotein of other coronaviruses, including severe acute respiratory syndrome (SARS) coronavirus (CoV) [35,36], SARS-CoV-2 [37,38,39], Middle East respiratory syndrome (MERS) CoV [40], mouse hepatitis virus (MHV) [41], porcine epidemic diarrhea virus (PEDV) [42], and transmissible gastroenteritis virus (TGEV) [43], through a variety of epitope-mapping approaches including computational structural analysis of receptor binding sites, in silico predictions, and phage display. Many protective and neutralising epitopes tend to be conformational in nature, and B-cell epitopes are often discontinuous with a characteristic residue length of 5–20 amino acids, with a shorter peptide spanning most of the epitope’s key functional residues [44]. It is generally believed that most of the identified linear antigenic determinants are contributory parts of conformational or discontinuous B-cell epitopes [45,46,47,48]. The strategy for the epitope identification of antibodies using peptide arrays was initially developed using linear peptide sequences [49]. More recently, the peptide-based Chemical LInkage of Peptides onto Scaffolds (CLIPS) epitope mapping technology was developed [50,51]. CLIPS involves the multiple cyclisation of linear peptides via reactions with chemical scaffolds, so that the peptide folds around the scaffold, restricting the flexibility and adopting a 3D conformation designed to present the appropriate order and configuration required to functionally reconstitute conformational epitopes found on native protein antigens [50,51,52]. As the nature of CLIPS presents peptides on a range of constructs, this increases the complexity of the arrays, due to their capacity to present a large number of peptides across a wide range of constructs and permit overlapping peptide sequences, allowing for high-resolution epitope mapping. Epitope mapping utilising peptide arrays has been a useful approach to identify immunodominant and sub-dominant epitopes for vaccine development in a range of viruses: Ebola virus [53,54], swine influenza virus [55], bovine herpesvirus-1 [56], equine arteritis virus [57], and porcine reproductive and respiratory syndrome virus [58].

The aim of this study was to identify shared and unique immunogenic epitopes of the S glycoprotein of three major circulating strains of IBV. We used CLIPS arrays based on the peptides from the S1 of IBV serotypes, QX, 4/91, and M41. The screening of the peptide arrays was performed with sera from chickens immunised with recombinant IBV, generated in two independent homologous and heterologous vaccination/challenge trials [20,59]. Due to the isogenic recombinant IBV backbone used for the expression of the S glycoprotein, this ensured that all other structural and non-structural proteins were similar and did not influence the induction of antibodies. This approach allows us to identify the linear and conformational epitopes present on the IBV S1 glycoprotein, and distinguish those which are shared amongst serotypes and those which are specific to certain serotypes.

## 2. Materials and Methods

### 2.1. Ethical Statement

All animal experimental protocols referenced here were carried out in strict accordance with the UK Home Office guidelines and under license granted for experiments involving regulated procedures on animals protected under the UK Animals (Scientific Procedures) Act 1986. The experiments were performed in The Pirbright Institute Home Office licensed (X24684464) experimental animal house facilities, and were approved by the animal welfare and ethical review committee under the terms of reference HO-ERP-01-1.

### 2.2. Homologous and Heterologous Vaccination/Challenge Trials: Sera for Epitope Mapping

The chicken sera used to screen the synthetic CLIPS peptide arrays were generated from two independent in vivo vaccination/challenge experiments of specific-pathogen-free (SPF) Rhode Island Red chickens with recombinant IBV (based on Beau-R backbone), as described in [20,59]. Briefly, in the first trial, the chickens were vaccinated with rIBV Beau-R expressing M41 S1 [BeauR-M41(S1)] or QX S1 [BeauR-QX(S1)], and then challenged with the homologous strain, M41 or QX [20,60,61]. All polyclonal sera used in the peptide screening were collected at 14 days post challenge (dpc), contained IBV-specific antibodies, and exhibited a degree of neutralising activity [20]. To permit the investigation of epitopes shared amongst IBV serotypes and elucidate their role in cross-protection, serum was also generated from a heterologous vaccination/challenge trial [59]. Briefly, the chickens were vaccinated with rIBV Beau-R expressing M41 S [BeauR-M41(S)] or 4/91 S [BeauR-4/91(S)], followed by a homologous or heterologous secondary vaccination with either M41 or 4/91, and then challenged with the heterologous strain QX [27,59,62].

### 2.3. Synthesis of CLIPS Peptide Arrays

The CLIPS peptide arrays were synthesised by Pepscan Presto BV (Lelystad, The Netherlands). Three different arrays were generated based on the S1 sequence of three economically important strains of IBV, M41-CK (Massachusetts serotype), 4/91 (793B serotype; UK), and QX-like (L1138). The 15-mer peptides, derived from the target S1 sequences, were positioned on the arrays, with the secondary structure and CLIPS technology used to inform positioning to simulate both linear and conformational epitopes. The linear 15-mer peptides were offset by one residue and additionally chemically constrained via the CLIPS technology into loop, helical, or coil constructs. The three IBV S1 peptide arrays contained helical (1564), coil (1553), linear (1574), and loop (1574) constructs with one array per serotype (Table 1).

### 2.4. Serum Screening of CLIPS Peptide Arrays

The CLIPS peptide arrays were incubated with chicken serum or S-specific mouse monoclonal antibodies (mAbs) (Table 2). Each individual serum sample was diluted to an optimal dilution (in range of 1:1000–1:20,000) in order to reduce background signal. All samples were diluted in PBS with 5% bovine-serum albumin (BSA) and horse serum (HS), and incubated on the peptide arrays overnight at 4 °C. High-stringency conditions, with the inclusion of both BSA and HS in the sample, blocking and wash buffer, were used in order to optimize signal-to-noise ratios in each independent experiment. After washing with PBS (5% BSA and HS), the peptide arrays were incubated with either HRP-conjugated goat anti-chicken IgY (H + L) (Southern Biotech, Birmingham, AL, USA) or HRP-conjugated mouse anti-chicken IgY (H + L) (Antibodies Online, ABIN2704014, Limerick, ME, USA) for 1 h at 25 °C. After washing with PBS (5% BSA and HS), the peroxidase substrate 2,2′-azino-di-3-ethylbenzthiazoline sulphonate (ABTS) and 20 µL/mL of 3% hydrogen peroxide were added. After 1 h, the colour development was measured and quantified with a charge-coupled device (CCD). The values obtained from the CCD camera ranged from 0 to 3000 mAU.

### 2.5. Quality Control Assessment of CLIPS Peptide Arrays

To validate the specificity of the synthesized CLIPS peptide arrays, expressing IBV peptides, two negative mAbs, CVI-TGEV-57.9 specific for the S protein of TGEV [63] and Herceptin™, were screened against the three IBV peptide arrays under conditions similar to those used for test serum samples. A panel of eight mAbs, which had previously been generated against the S1 region of IBV D207 strain (Table 2), some of which had virus neutralization activities and whose recognition sites were distributed across the defined antigenic regions of S1 from IBV D207 strain [17,21,22], were used to evaluate the specificity of the three CLIPS peptide arrays based on IBV S1 of M41, 4/91, and QX.

### 2.6. Analysis of Predicted Epitope Sequences

Intensity profiles were examined for each individual chicken serum sample, tested on all three (M41, QX, and 4/91) IBV S1 peptide arrays, and screened against all four types of constructs: linear, helical, coil, and loop. The level of non-specific background staining was determined on an individual sample level, with a common range of 0–200 AU. Peptides associated with a peak in the absorbance traces were recorded. The intensity of the antibody binding peaks in the absorbance traces varied at an individual sample level. Consequently, the levels of non-specific background staining displayed by individual serum samples were taken into consideration, and clear discernible peaks in intensity were evident. The levels of conservancy of peptides selected by antibody binding across serum samples were assessed. The epitopes were classified as “linear” or “conformational”, depending on the type of peptide construct associated with the peak in absorbance. The peptide binding profiles of serum samples were compared to identify regions of similarity and differential binding. Heat map profiles of the binding intensity were constructed using the ‘pheatmap’ function (pheatmap package) in R version 3.4.1. Dendrograms between serum samples were calculated using the Pearson correlation clustering algorithm, based on the average pairwise distance between all points of each data set and scaled to the intensity of rows.

### 2.7. Linear Peptide ELISA

To validate the antibody binding of the three selected peptide sequences on an individual capacity, the recognition of linear peptides was assessed in a synthetic peptide ELISA. Biotinylated synthetic peptides were synthesized (Eurogentec, Seraing, Belgium) based on the binding specificity of the chicken sera with the peptide arrays. The three selected peptides were diluted to 10 µg/mL, and incubated on streptavidin pre-coated plates (Thermo Fisher, Waltham, MA, USA.) overnight at 4 °C. The plates were then washed four times with PBST (0.05% Tween-20). Serum samples were diluted 1:10 in PBS, applied to the plates, and incubated for 2 h at 37 °C. The plates were re-washed with PBST and mouse anti-chicken IgY (Clone G-1) (Southern Biotech, γ-chain specific), diluted 1:1000, was applied and incubated for 1 h at 37 °C. The plates were washed with PBST and HRP-conjugated goat anti-mouse IgG1 tertiary antibody (Southern Biotech), diluted 1:2000, was applied and incubated for 1 h at 37 °C. Finally, the plates were washed and developed with 3,3′,5,5′-Tetramethylbenzidine (TMB) substrate (Thermo Fisher), and the reaction was stopped with 2 M H_2_SO_4_ and read at 450 nm on a microplate reader.

### 2.8. Conservation of Epitope Sequences and Structural Modelling

A bioinformatic analysis to assess the conservation of the identified epitopes amongst IBV serotypes was performed. Sequence logos were used to determine the sequence conservation of the identified epitopes, amongst the three IBV strains studied here, QX, 4/91, and M41, using AliView v1.25 [64]. A sequence logo provides a comprehensive description of the level of conservancy rather than consensus sequences, and can reveal significant structural and functional features of the epitopes. The structural analysis was performed by using the co-ordinates of the IBV-M41 spike (PBD ID 6CV0) [15]. The structure was visualized and manipulated using the program PyMOL 1.8.2.3.

**Table 2:** Serum samples and antibodies used for epitope mapping.

Group	Antibody Type	Antibody Raised Against	Antigenic Region ^e^	Challenge Virus	Reference
Homologous	Polyclonal	M41(S)	N/A	M41	[20]
		M41(S1)			
		QX(S1)		QX	
Heterologous	Polyclonal	M41(S) ^a^	N/A	QX	[59]
		M41(S) and 4/91(S) ^b^			
		4/91(S) ^c^			
Mock	Polyclonal	N/A	N/A	N/A	[20]
Mock/challenge	Polyclonal	N/A	N/A	M41 QX	[20,59]
Monoclonal antibodies	CVI-IBV-48.1	IBV strain D207 S1 ^d^	A/B (#N)	N/A	[21,22,65]
	CVI-IBV-48.2		C (#N)		
	CVI-IBV-51.2		ND		
	CVI-IBV-52.1		E (#N)		
	CVI-IBV-62.1		A (#N)		
	CVI-IBV-62.8		D (#N)		
	CVI-IBV-69.1		A (#N)		
	CVI-IBV-69.3		E/F		

^a, c^ Chickens received a homologous secondary boost (which was the same as the primary vaccination). ^b^ Chickens received a heterologous secondary boost (which was different to the primary vaccination). ^d^ Panel of monoclonal antibodies used for array affinity. (#N denotes neutralising activity). ^e^ Antigenic region of IBV M41 S according to [17,21,22]. ND denotes not determined.

## 3. Results

### 3.1. Construction of the IBV S1 Peptide Arrays

Constrained linked peptide screening (CLIPS) oligopeptides were synthesised using various scaffold combinations on a solid surface to produce three individual peptide arrays to permit the probing of antibody binding in a high-throughput epitope mapping system. These CLIPS were used to produce three peptide arrays based on the known S1 sequence of IBV strains; M41-CK, 4/91 (UK), and QX (L1148), containing 2093, 2090, and 2082 oligopeptides, respectively. Using the BLOSUM62 similarity matrix, the predicted S1 amino acid sequences of M41-CK and 4/91 (UK) are 81.7% identical and 89% similar, those of M41-CK and QX (L1148) are 80.8% identical and 89% similar, and those of 4/91 (UK) and QX (L1148) are 83.9% identical and 91.2% similar (Supplementary Figure S1). These complex CLIPS peptide arrays were expected to produce different arrangements of the component peptide fragments, leading to conformational-constrained epitopes similar to those present on the surface of IBV S trimers. For screening purposes, the CLIPS peptides are bound to solid supports where they can fold, or become constrained in higher order structures such as β-sheets, α-helices, and looped structures. Peptide arrays covering the S1 subunit of the IBV S glycoprotein, from three strains, M41, QX, and 4/91, were used to map immunologically important regions on the protein, in an attempt to identify antigenic regions on the S1 that are shared amongst IBV serotypes. To mimic linear and conformational epitopes on the surface of the trimeric glycoprotein, series of CLIPs were synthesised, and each peptide was displayed on four types of constructs. The construct type and number of CLIPS peptides included in all peptide arrays are presented in Table 1.

As a validation step to assess the specificity of the three peptide arrays based on the S1 sequence of IBV strains, M41, QX, and 4/91, the arrays were screened using eight mAbs, which had been raised against IBV strain D207 [17], and selected based on their neutralising activity and distribution of their recognition sites throughout the S1 (Table 2). The levels of amino acid similarity between the S1 sequence of D207 and that of M41, 4/91, and QX are 77.43%, 78.44%, and 76.58%.

**Figure 1:**
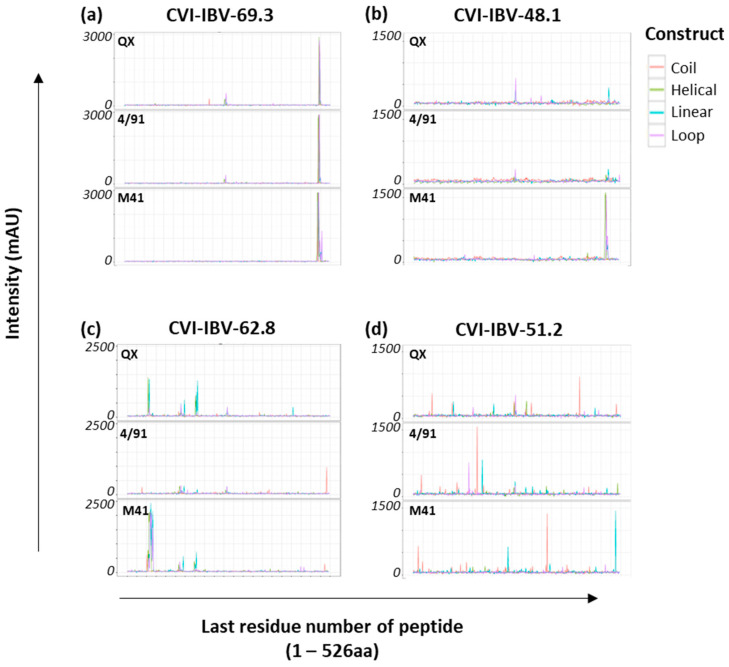
Recognition of IBV S1 peptide arrays with a panel of IBV mAbs. Representative example of epitope mapping of four anti-IBV S1 monoclonal antibodies, (**a**) CVI-IBV-69.3, (**b**) CVI-IBV-48.1, (**c**) CVI-IBV-62.8, and (**d**) CVI-IBV-51.2, screened against all three IBV S1 peptide arrays. Individual IBV S1 peptide arrays are labelled according to the specific S1 sequence from which the CLIPS peptides were derived. The x-axis is the position of the last residue of a peptide with respect to the appropriate IBV S1 sequence. Optical density signals obtained via CCD measurement are given in milli-absorbance units (mAU) for all CLIPS peptides. All intensity profiles were smoothened using moving average function with window of three, and are depicted for all four construct types displayed on each S1 array, namely, coil, helical, linear and loop, which are colour coded as depicted in the key for construct.

Three of the eight mAbs tested yielded conclusive binding profiles, and epitope candidates were proposed (Figure 1a–c). The remaining mAbs (*n* = 5) (Table 2) also bound to the peptide arrays, albeit with lower intensity and multiple peaks evident, as depicted by the trace recorded for CVI-IBV-51.2 (Figure 1d). One caveat is that some of the mAbs used for screening here have been shown to lose the ability to bind to virus or S1 following de-glycosylation, showing the importance of glycosylation and protein conformation [22]; therefore, it is likely that the five mAbs shown here to have lower intensity will require glycosylated targets or a native protein conformation to enhance the level of antibody recognition. However, three antibodies from the panel had high affinity for the IBV peptide arrays (Figure 1a–c). Monoclonal antibodies CVI-IBV-69.3 (Figure 1a) and CVI-IBV-48.1 (Figure 1b) recognise overlapping regions near the C-terminus of IBV S1. CVI-IBV-48.1, in particular, had high affinity for epitopes presented on the S1 M41 array with a defined sequence, VNQQFVVSGGKL, and only a weak positive residual interaction was observed with peptides derived from 4/91 and QX at the corresponding locations (Figure 1b).

The recognition site for CVI-IBV-69.3 was conserved across all three IBV S1 peptide arrays (Figure 1a); however, there were two amino acid differences in the defined peptide sequence: QQFVVSGGK[K/N][L/I]VGIL. CVI-IBV-62.8 mAb recognised multiple CLIPS peptides as it strongly bound peptides presented on the M41 S1 peptide array with core sequence VGTIHGGRVV and the corresponding region NNAGSAHQCTVGVIK on the S1 QX array, near the N-terminus of the S1 sequence, whereas there was no recognition of peptides within this region on the S1 4/91 array (Figure 1c). Additional lower intensity binding peaks were observed with peptides containing sequences TTDVTSAGVYFK (on both the QX S1 and M41 S1 peptide arrays) and TVSV[S/A]KYPXFKS[F/L]QC on the 4/91 S1 peptide array. Following these steps, the three IBV S1 peptide arrays generated were shown to have specificity for antibody binding and were subsequently screened with the polyclonal chicken sera from both the homologous and heterologous vaccination/challenge trials.

### 3.2. Array Screening with Sera from Homologous rIBV-Vaccinated Chickens

**Figure 2:**
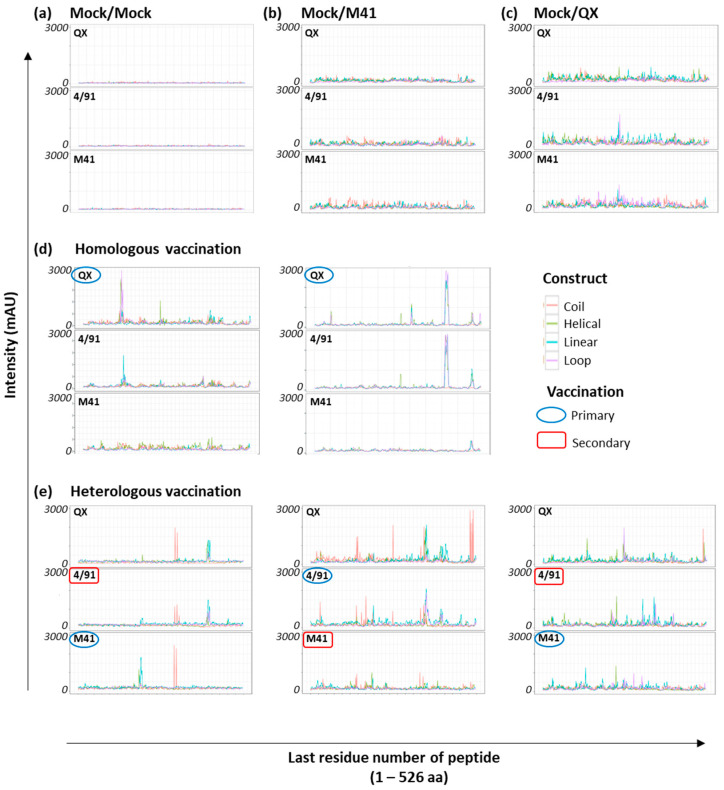
Antibody binding of IBV S1 peptide arrays. Representative examples of epitope mapping of experimentally derived polyclonal serum against the three IBV S1 peptide arrays: (**a**) Mock/Mock = non-vaccinated/non-challenged, (**b**) Mock/M41 = non-vaccinated/M41 challenge, (**c**) Mock/QX = non-vaccinated/QX challenge, (**d**) two individual serum samples from the homologous vaccinated/challenged chickens, and (**e**) three individual serum samples from the heterologous vaccinated/challenged chickens. The three IBV S1 peptide arrays are labelled according to the specific S1 sequence from which the CLIPS peptides were derived. The x-axis is the position of the last residue of a peptide with respect to the appropriate IBV S1 sequence. Optical density signals obtained via CCD measurement are given in milli-absorbance units (mAU) for all CLIPS peptides. The intensity traces are depicted for all four construct types (coil, helical, linear, and loop) displayed on each individual S1 peptide array, colour coded as depicted.

To elucidate the profile of recognition of the CLIPS peptides on the IBV S1 peptide arrays, individual sera from homologous vaccinated/challenged chickens, collected after challenge with homologous wild-type virus [20], were used to screen the arrays (*n* = 20). There were two main aims in these screening experiments: (1) assess the antibody specificity of the polyclonal serum for CLIPS-peptides, and (2) identify if there are serotype-specific epitopes recognised on the S1 of the three IBV strains under investigation. In addition to the sera from homologous vaccinated/challenged chickens, the three IBV S1 peptide arrays were also screened with pools of sera, collected at 14 dpc, from non-vaccinated/non-challenged chickens (“Mock/Mock”) (pool of *n* = 10 samples) and non-vaccinated/challenged chickens for both viruses, (Mock/QX or Mock/M41) (pool of *n* = 10 samples per challenge group). Intensity profiles for all homologous serum samples were recorded against each type of construct displayed on the three IBV S1 peptide arrays following screening with pooled Mock/Mock and Mock/challenge only sera (Figure 2a–c) and the individual sera from homologous vaccinated/challenged chickens (Figure 2d).

The screening of the IBV S1 peptide arrays with the pool of age-matched non-vaccinated/non-challenged sera (“Mock/Mock”) showed very little background reactivity of the CLIPS peptides (Figure 2a). In comparison, the 14 dpc sera from non-vaccinated/challenge only controls displayed weak staining with no discernible peaks, across all three of the arrays (Figure 2b,c). The screening of the IBV S1 peptide arrays with individual sera from the homologous vaccinated/challenged chickens (Figure 2d) revealed profiles of antibody binding to CLIPS peptides with clearly defined peaks for 11 out of the 20 samples screened. The remaining nine serum samples from the homologous vaccinated/challenged chickens did not give rise to clear, defined epitope profiles. This was either due to an elevation in baseline background binding or a lack of recognition of CLIPS peptides epitope mimics, making it difficult to distinguish specific binding.

**Figure 3:**
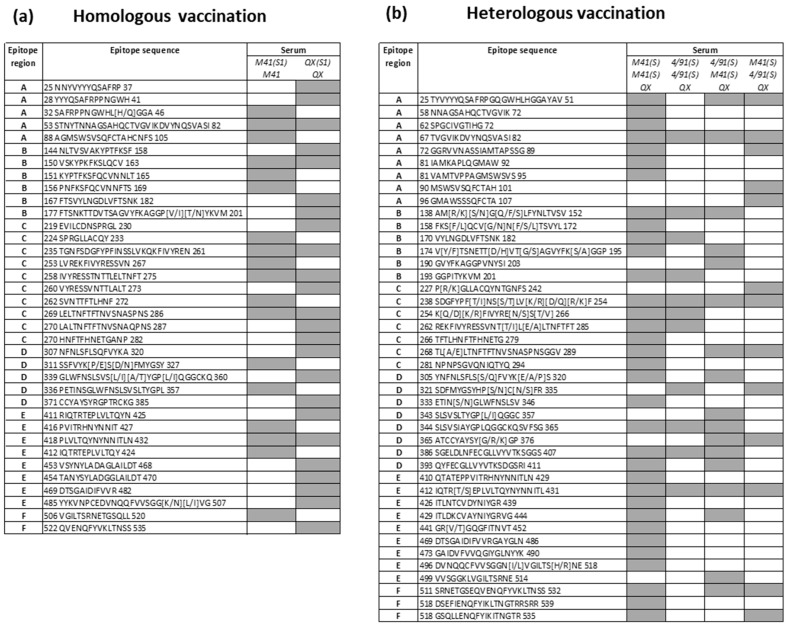
Recognition of selected putative epitopes across all three IBV S1 peptide arrays. Polyclonal sera classified according to recombinant IBV vaccination trial: (**a**) homologous vaccination/challenge and (**b**) heterologous vaccination/challenge. Shaded boxes indicate the recognition of peptide sequence by serum from the specified vaccinated group. Vaccination and challenge virus received are depicted in italics in the table vertically in the “Serum” column as follows: Homologous vaccination (Vaccination–Challenge) and Heterologous vaccination (Vaccination–Boost–Challenge). Epitopes were assigned to epitope binding regions (termed “A–F”, split by 100aa due to overlapping individual epitope sequences), and the amino acid position was given at the start and end of the peptide sequence.

Collectively, there were 36 epitope sequences recognised by polyclonal sera from the homologous vaccinated/challenged chickens (Figure 3a), with 12 and 18 sequences unique to the M41 S1 and QX S1 peptide arrays, respectively. Across the peptide arrays, there were six sequences which were shared (i.e., recognised by both homologous serum sets on both the M41 and QX S1 arrays) (Figure 3a). As there was a degree of overlap between some of the identified individual epitope sequences, it was necessary to consider epitope regions; for this purpose, the S1 sub-unit could be split into 100 aa epitope regions (termed “A–F”), covering the N-terminal and C-terminal domains of the sub-unit.

### 3.3. Identification of Shared Epitopes by Screening with Heterologous rIBV Sera

A second screening experiment of the IBV S1 peptide arrays was performed with the polyclonal sera collected at 14 dpc from the heterologous vaccination/challenge experiment [59] (*n* = 38). In a similar manner to the homologous serum screening, two additional pools of age-matched serum controls were used (*n* = 10 birds per pool): (1) non-vaccinated/non-challenged group and (2) non-vaccinated/QX challenge only (Table 2). Epitope mapping was successful for 23 out of the 38 individual sera from heterologous vaccinated/challenged chickens, with a representative example of the intensity traces shown in Figure 2e. The level of background binding was variable across the sera from heterologous vaccinated/challenged chickens, with some samples showing higher background signals across all three IBV S1 peptide arrays and against the four construct types displayed on these arrays. As a result, 15 of the heterologous samples screened did not give rise to clear, defined epitope profiles, either due to an elevation in baseline or a lack of strong recognition of epitope mimics. From the 22 individual sera from heterologous vaccinated/challenged chickens, there were 42 epitopes identified across the IBV S1 amino acid sequences, with a high degree of overlap between the four different heterologous vaccinated/challenged groups (Figure 3b). The highest proportion of epitope sequences was identified in the homologous boosted group, BeauR-M41(S)/BeauR-M41(S), at 34 epitopes, compared to the other homologous boosted group, BeauR-4/91(S)/BeauR-4/91(S), (11 epitopes) or the other two heterologous boosted groups, BeauR-4/91(S)/BeauR-M41(S) and BeauR-M41(S)/BeauR-4/91(S), with 17 and 13 epitopes identified, respectively (Figure 3b). As all of these vaccinated groups received the same heterologous challenge with wild-type QX, 50% (21/42) of the epitopes recognised were shared across the IBV strains investigated here, i.e., identified by more than one vaccination group, and the other 50% were identified by serum from a single vaccination group (Figure 3b).

Collectively, a total of 78 epitope sequences were identified through screening the IBV S1 peptide arrays with the panel of polyclonal sera from both the homologous and heterologous vaccination/challenge experiments (Figure 3a,b).

### 3.4. Clustering to Investigate Epitope Binding Profiles

**Figure 4:**
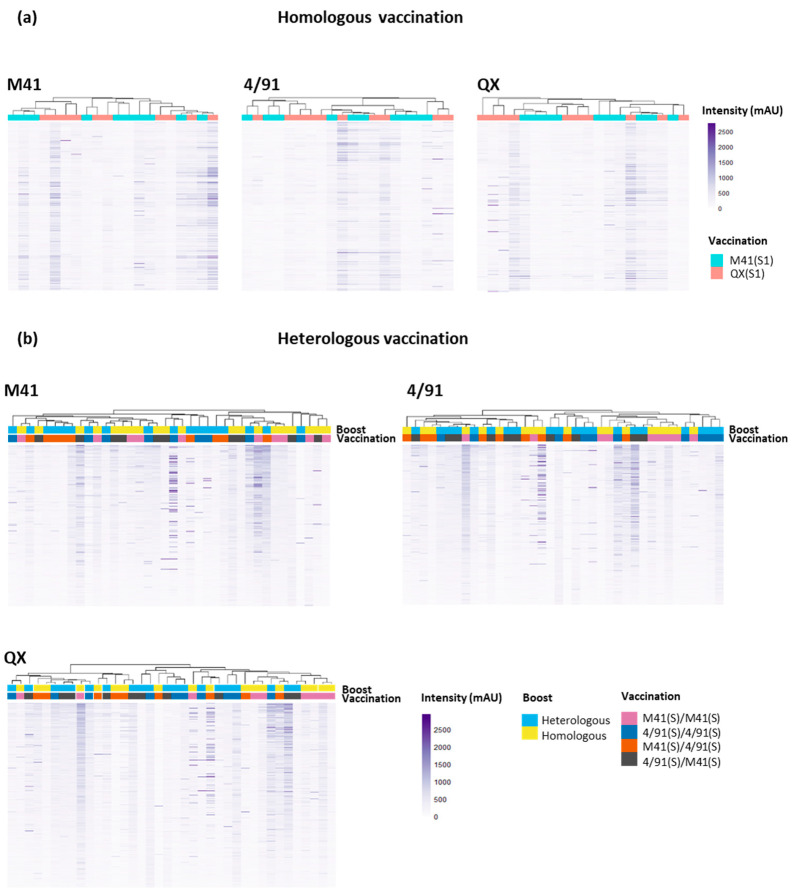
Heat maps showing the recognition of peptide sequences by individual 14 dpc serum samples by binding to the three IBV S1 peptide arrays; QX, 4/91, and M41. Panel (**a**) shows individual sera from homologous vaccinated/challenged chickens (*n* = 20) binding to serotype-specific epitopes across all three IBV S1 arrays. Panel (**b**) displays the profiles of individual sera from heterologous vaccinated/challenged chickens (*n* = 38) binding to IBV S1 peptide arrays. All serum samples were collected at 14 dpc. Putative epitope sequences are shared across IBV serotypes. Dendrograms between serum samples were calculated using Pearson correlation, based on the average pairwise distance between all points of each data set and scaled to the intensity of the rows. The x-axis represents an individual serum sample. The y-axis represents a single peptide sequence.

All of the sera samples from vaccinated chickens (including samples for which no defined epitope profiles were distinguished, i.e., “unmapped”) were subjected to a clustering analysis to investigate if any similarities or patterns between the immune recognition of individual samples were evident (Figure 4).

In addition, this clustering analysis was performed with an aim towards the identification of antibody binding regions on the three S1 peptide arrays which were unique or shared amongst IBV serotypes. A cluster analysis of the profiles of antibody binding to the peptide arrays was also performed for the “Mock/Mock” and “Mock/Challenge” controls, showing little background binding in the “Mock/Mock” and some intensity peaks in the “Mock/Challenge” serum (Supplementary Figure S2). The binding profiles of the homologous QX serum on both the M41 S1 and 4/91 S1 peptide arrays showed strong cross-reactivity, depicting a number of shared epitopes displayed on both the M41 and 4/91 S1 sub-units, and a limited number of unique areas (Figure 4a, Supplementary Figure S3). The cluster analysis of the heterologous challenged serum permitted a deeper investigation into antigenic regions on the IBV S1 subunit shared amongst serotypes. A high degree of overlap was revealed between IBV S1 epitope regions recognised by heterologous challenged serum, with no clear clustering into their vaccination groups (Figure 4b). The vaccination order, or if a homologous or heterologous secondary boost was given, did not appear to induce a difference in the antibody binding profile, as there was no definitive grouping based on these criteria (Figure 4b, Supplementary Figure S4). Undertaking this clustering analysis allowed further investigation to determine whether the issues with serum samples that did not give rise to clear, defined epitope profiles (*n* = 10, homologous vaccination/challenge and *n* = 15 heterologous vaccination/challenge) was either due to lower affinity for the CLIPS or lack of recognition of the epitope mimics. From the comparison of the epitope binding profiles, it appears that these sera had lower affinity for the CLIPS and did not reach the same intensity as the other sera from vaccinated chickens (Figure 4a,b).

### 3.5. Accessibility and Location of IBV Spike Epitope Regions

**Figure 5:**
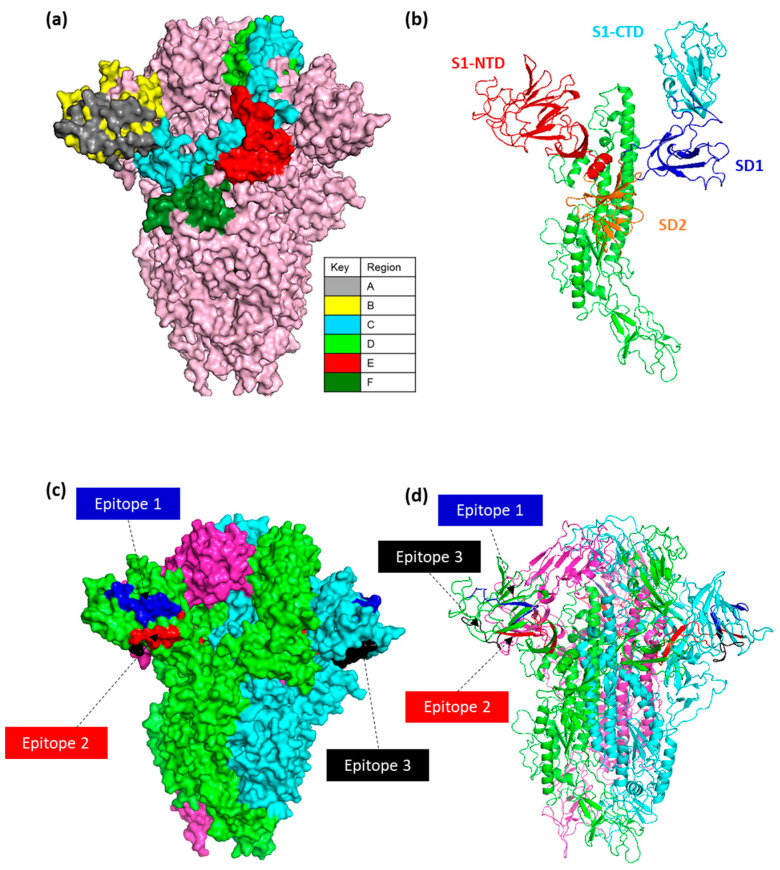
IBV spike structural modelling. (**a**) A surface model representation of the IBV M41 spike trimer is shown in light pink. Epitope regions (A–F) of one of the S1 monomers are coloured as per key. (**b**) Structural domains of IBV M41 spike highlighted on one monomer, S1-NTD (19–237aa), S1-CTD (269–414aa), SD1 (248–269aa, 414–492aa), and SD2 (237–248aa, 492–567aa). (**c**) Trimeric surface and (**d**) structural model of IBV M41 spike with three selected epitopes highlighted with colour coding as per Epitope 1 (Blue), Epitope 2 (Red), and Epitope 3 (Black). The model was built using structural homology based on the cryo-EM of IBV M41 spike and structural domains annotated as stated in [14].

Structural modelling of the IBV S glycoprotein was conducted to visualize the expected location of both the selected epitopes and antibody binding regions identified during the screening of the CLIPS peptide arrays (Figure 5a–d). The structural model used here was built using structural homology based on the cryo-EM of the pre-fusion IBV M41 S (PDB id: 6CV0) [15].

The overlapping epitopes identified were located throughout the N-terminal and C-terminal domains of the S1 subunit. As a result, epitope binding regions were assigned split into 100 aa regions) and termed “Epitope regions A–F”. Analysis showed that 55% (43/78) of the epitope sequences identified were regions A–C. Epitope regions A and B are located across the S1-NTD (19–237 aa), speculated to contain the receptor binding domain (RBD) and the hypervariable regions HVR1 (38–67 aa) and HVR2 (99–141 aa) [16,18,65,66,67], and characteristically are presented as β-sheets (Figure 5a,b,d). Region C overlaps the S1-NTD (200–269 aa) and S1-CTD (269–414 aa), and partially into the S1-NTD across 200–237 aa (Figure 5a,b). Regions D–F are located in the subdomains (SD1 and SD2), and epitopes were found in higher order structure of β-sheets and α-helices (Figure 5a–d).

### 3.6. Conservancy of Selected Epitope Candidates amongst IBV Serotypes

**Figure 6:**
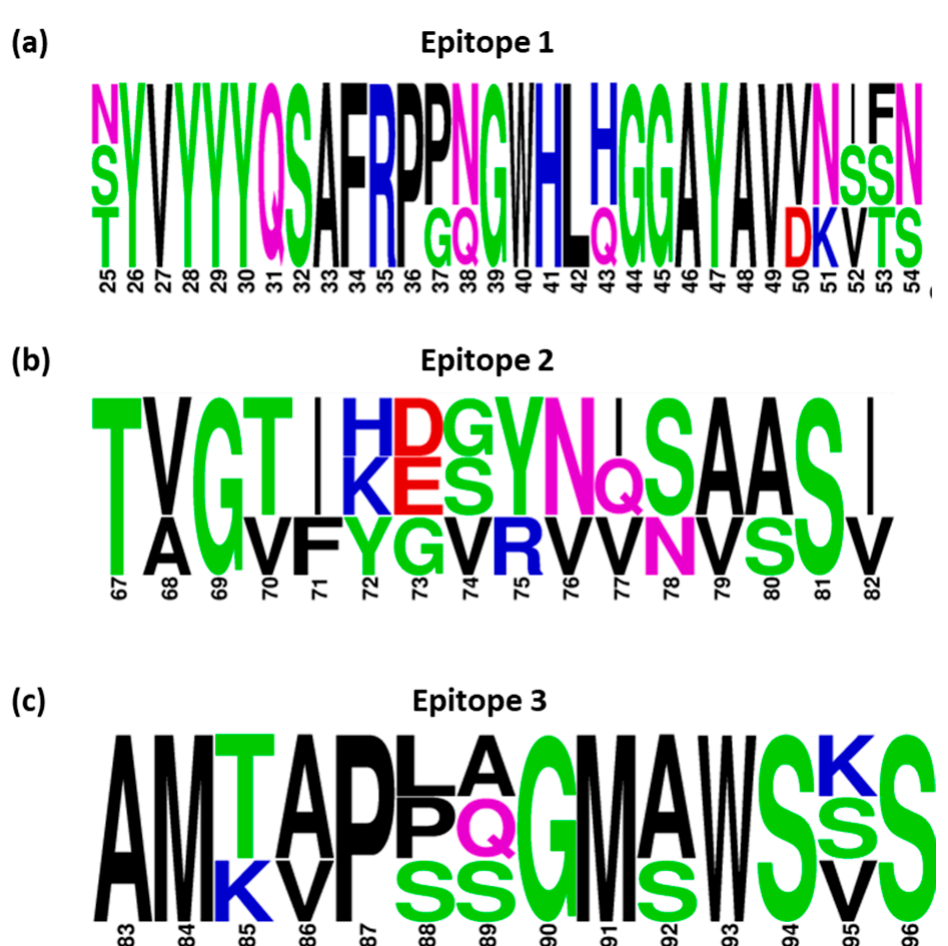
Conservation of identified peptide epitopes on the spike glycoprotein of IBV strains, M41, QX, and 4/91. Logo analyses are shown for the frequency of each amino acid within each of the three epitopes in spike from selected IBV strains, (**a**) Epitope 1, (**b**) Epitope 2 and (**c**) Epitope 3. The height of the letter indicates the frequency of the individual amino acid residue at that site. The residue position in the block is shown on the X-axis, and the information content is shown on the Y-axis. The default colour scheme displaying different amino acids according to their different chemical properties is as follows: polar amino acids (G, S, T, Y,) coloured with green, (C, Q, N) coloured with pink, basic (K, R, H) with blue, acidic (D, E) with red, and hydrophobic (A, V, L, I, P, W, F, M) with black.

Out of the 78 epitope sequences identified (Figure 3), 3 sequences were selected based on their location and the following criteria for further investigation: (1) spatially separated, (2) located within the S1-NTD (likely to contain the RBD), and (3) with any proximity to the hypervariable regions, HVR1 (38–67 aa) and HVR2 (91–141 aa), that is associated with virus-neutralising activity. The epitope sequences selected were as follows: ^25^YVYYYQSAFRPPNGWHLQGGAYAVVNSTN^54^ (Epitope 1—QX), ^67^TVGVIKDVYNQSVASI^82^ (Epitope 2—QX), and ^83^AMTVPPAGMSWSVS^96^ (Epitope 3—4/91). Sequence logos were used to assess the conservation of individual amino acids in each identified epitope, with the height of each letter indicating the proportion of sequences that contain the residue at that site (Figure 6).

The majority of residues in ^25^YVYYYQSAFRPPNGWHLQGGAYAVVNSTN^54^ (Epitope 1) were shared amongst IBV serotypes QX, 4/91 and M41, but there were amino acid substitutions at positions 25, 37, 38, 43, 50, and 51–54 (Figure 6a). The epitope denoted as ^83^AMTVPPAGMSWSVS^96^ (Epitope 3) was also shared amongst serotypes, with some degree of variation evident in residues and where substitutions occurred (positions 85, 86, 88, 89, 92, and 95); there were changes in amino acid hydrophobicity when serine was in these positions (Figure 6c). In contrast, ^67^TVGVIKDVYNQSVASI^82^ (Epitope 2) appeared to be serotype-specific with a high level of residue variability, evident from the equal weighting of residues across the peptide sequence (Figure 6b). For the shared epitopes, Epitope 1 and Epitope 3, the profile of binding to these epitopes displayed on multiple constructs across all three IBV S1 arrays (QX, 4/91, and M41), with analysis using the clustering methods described earlier, reinforced the conservancy of these specific sequences across IBV serotypes (Figure 6a–c).

**Figure 7:**
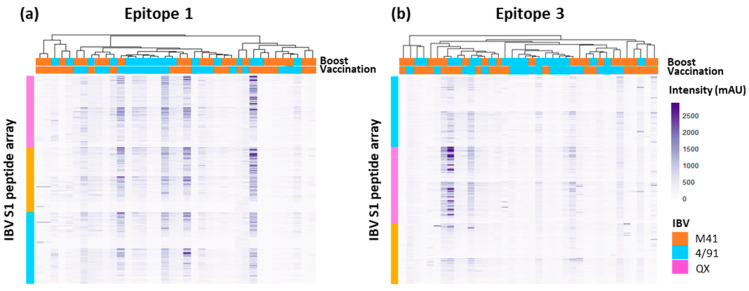
Heat maps showing the recognition of selected peptide sequences by individual 14 dpc serum samples from heterologous vaccinated/challenged chickens. All heatmaps show the selected epitope sequence displayed on multiple constructs across the IBV S1 peptide arrays: panel (**a**) Epitope 1 and (**b**) Epitope 3. The y-axis represents a single construct-specific peptide, displaying the selected epitope sequence across all three IBV S1 peptide arrays. The x-axis represents an individual serum sample. Serum samples included individual mapped and unmapped (classified with background deemed too high to identify clear peaks in intensity traces) samples collected at 14 dpc. Dendrograms between serum samples were calculated using Pearson correlation, based on the average pairwise distance between all points of each data set and scaled to the intensity of the rows.

The cluster analysis of the binding profiles of the individual sera from heterologous vaccinated/challenged chickens to IBV-S1 derived peptides containing Epitope 1 or Epitope 3 shows similarity in the patterns of binding across all three IBV S1 peptide arrays (Figure 7a,b). This further supports the conservation of both Epitope 1 and Epitope 3 across the three IBV serotypes, M41, 4/91, and QX, investigated here.

### 3.7. Assessment of Selected Epitopes 1–3 for Vaccine Candidate Potential

**Figure 8:**
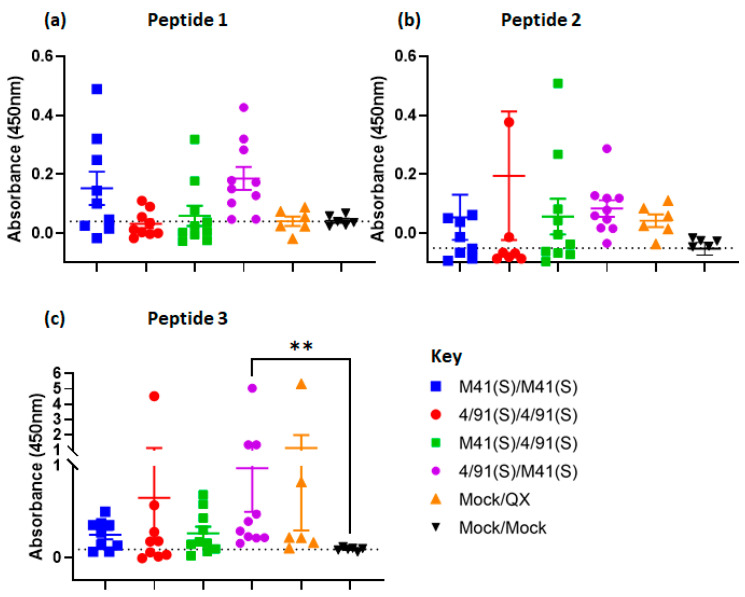
Recognition of synthetic peptides (representing epitopes 1–3) by sera from heterologous vaccinated/challenged chickens. Serum anti-peptide titres were assessed by a peptide-based ELISA, (**a**) Peptide/Epitope 1, (**b**) Peptide/Epitope 2 and (**c**) Peptide/Epitope 3. The sera from heterologous vaccinated, Mock/QX, and Mock/Mock chickens were diluted 1:40 and corrected for background absorbance before the determination of the recognition of peptides. Individual values and the mean (±standard error of mean) from each group (*n* = 10) were corrected for background absorbance, and include three technical replicates/bird. The dashed line indicates the cut-off threshold which was calculated on the mean value obtained for the Mock/Mock group. Statistical differences between groups were evaluated using Kruskal–Wallis with correction for Dunns’s multiple comparison tests to Mock/Mock, where ** denotes a significant difference, *p* < 0.05.

Biotinylated linear peptides of Epitopes 1–3 were synthesised and used in an ELISA to validate the recognition of epitopes by sera from vaccinated chickens and evaluate their potential as vaccine candidates (Figure 8a–c).

Epitope 1 was recognised by sera from all vaccinated groups (Figure 8a), although recognition was highly variable within vaccinated groups and non-significant compared to Mock/Mock controls (Figure 8a). For Epitope 3, the only vaccinated group to show significantly higher titres compared to the Mock/Mock control was the heterologous BeauR-4/91(S)/BeauR-M41(S) (Figure 8c, *p* < 0.05), with the recognition of the peptide being highly variable within the other vaccinated groups and the majority of serum samples having low to moderate binding. This pattern of low binding was also evident against Epitope 2, with no significant differences detected across the vaccinated groups in comparison to the Mock control (Figure 8b).

## 4. Discussion

In an attempt to address continual issues surrounding cross-protection with IBV vaccination, the focus here was on the identification and characterisation of immunogenic epitopes displayed on the S glycoprotein of highly prevalent and economically important strains of IBV. Sera generated in two rIBV vaccination trials, a homologous challenge [20] and a heterologous trial [59], provided a serum panel to screen epitope arrays of the S1 of IBV serotypes M41, QX, and 4/91. This is the first study demonstrating the application of screening CLIPS arrays in avian immunology as a tool for mapping the antigenic regions of the S1 of IBV serotypes M41, QX, and 4/91. To reveal epitopes that might be associated with cross-protection, sera from chickens vaccinated with rIBV were screened with the peptide arrays. The rIBVs used were isogenic, only differing in the S glycoprotein, ensuring that only antibodies raised against epitopes on the S should differ between chickens. The screening of serum samples from the rIBV vaccination experiments resulted in the detection of 78 highly immunogenic fragments in the S1 sequences. When these fragments were mapped onto the structural model of the IBV M41 S, they defined six overlapping epitope binding regions. Three peptide sequences were selected for further validation, and all of the selected epitopes were recognised to varying degrees by experimentally derived immune chicken serum from the rIBV vaccinated groups.

Epitope identification in the S glycoprotein across IBV serotypes has been an area of intrigue for researchers for the past 40 years [17,19,67,68,69,70]. Due to the continual failure to develop effective broad cross-protective vaccines, it remains a highly active area of research [71,72,73,74,75,76]. Over these research studies, many different approaches to identify epitopes have been undertaken, e.g., in silico prediction [69,73], raising and screening monoclonal antibodies [21,67,68], and and phage display peptide-mimetic screening [76]. Some researchers have generated synthetic linear peptides and screened these via ELISA [69,70,71,72,74]; however, a limitation of this approach is that it cannot identify conformation-dependent epitopes that may represent important antigenic determinants of the S protein of IBV. The identification of conformational epitopes is particularly difficult due to the fact that antibody–antigen complexes form with proteins in their native structure. The approach used here overcomes these limitations with CLIPS peptide arrays displaying multiple-scaffolds, constrained to mimic native α-helices and β-sheets in the tertiary structure, allowing an in-depth study of key interaction sites in proteins [50]. It is postulated that 90% of epitopes recognised by B-cells are conformational epitopes, and neutralising antibodies are generally produced against B-cell conformational epitopes [77,78]. CLIPS peptide arrays have proven to be a useful tool in deciphering both linear and conformational epitopes displayed on other viral surface antigens and recognised by B-cells, illustrated with HA of the Influenza A virus [79] and the glycoprotein of the Ebola virus [53,54].

We identified a large panel of both serotype-specific and shared overlapping epitopes displayed throughout the S1 of IBV M41, QX, and 4/91, and the presence of multiple immunogenic regions on the IBV S1 has been seen previously [17,21]. In 1987, Niesters et al. [67] found five epitopes on the S of IBV M41, two of which predominantly overlapped and one of which was serotype-specific and neutralising. The cross-reactive S1 epitope, ^240^GYNYGNFSDGFYPFTN^255^, shown to be conformationally independent and in close proximity to the S1/S2 junction [80,81], was also identified using the screening CLIPS arrays here.

Two studies focused on the in silico predictions of antigenicity and hydrophobicity of the S1 of IBV serotypes M41 and 793B, and revealed a panel of both B-cell and T-cell restricted epitopes, of which the 7/20 (M41) 5/17 (CR88) and 5/15 (793B) epitopes, respectively, matched our generated data [73,82]. In our study, we concentrated solely on the identification of B cell epitopes. Nevertheless, some of the epitopes we identified matched immunogenic epitopes predicted to be associated with cytotoxic T-lymphocyte (CTL) activity [70,74,75]. In addition, two serotype-shared B cell epitopes, ^25^TYVYYYQSAFRPGQGWHLHGGAYAV^51^ and ^305^YNFNLSFLSSFVYKESDFMYGSYHPSCSFR^335^, subsequently shown in induce neutralising antibodies, were also conserved between these three data sets [70,74]. Several attempts to utilise these epitopes in a vaccine construct, with or without the inclusion of immunogenic N protein epitopes, showed that the formulation of a multi-epitope DNA vaccine using these CTL and B-cell epitopes induced strong humoral and cell-mediated responses to control IBV challenge, characterised by viral neutralization and CD8 T-cell proliferation assays [70,75,83]. However, there was no direct assessment of the CTL response, which is of paramount importance for controlling early IBV infection and limiting viral replication [84]; protection against the heterologous challenge was also not demonstrated [70].

Interactions between the S1 and S2 subunits are critical for the conformation and efficient fusion of IBV S to host cells, as a single amino acid change in the S2 may influence the secondary structure [23]. This is further compounded by our previous findings with rIBV BeauR-M41(S), which was associated with the highest level of ciliary protection compared to Beau-R expressing M41 S1 or S2 sub-units alone [20]. It is important to note that it is possible that there are epitopes on S2 that are associated with a broad-spectrum of neutralising activity. However, there is still insufficient evidence that S2 epitopes alone can be cross-protective, as the recognition of linear S2 epitopes was not correlated with serum antibody titres or neutralising activity [71]. In addition, the prospective S2 epitope ^8^NCPYVSYGKFCIKPDGSIST^27^, identified by Ignjatovic and Sapats [72], was shown to be poorly conserved across serotypes, and synthetic epitopes spanning the IBV S2 were not recognised across multiple IBV serotypes [69]. Collectively, whilst the S2 has importance in controlling the quaternary structure of the spike, it is still not confirmed if there are specific S2 epitopes associated with a broad-spectrum protective response against IBV.

The publication of the cryo-EM structure of M41 [15] allows the visualisation of the distribution and location of the selected epitope sequences in tertiary protein structures and the assessment of accessibility. Logically, the accessibility of epitopes found on the native protein may influence the presentation and affinity of recognition by antibodies. Some of the mouse mAbs used to validate the arrays showed a lower binding intensity and may bind to less conserved parts of the spike protein in the different IBV strains or require glycosylated targets [22]. Using chicken sera, we identified a panel of epitopes, some of which are presented on the surface of the S glycoprotein, within the receptor-binding domain or at the S1/S2 interface. Immunogenic epitopes found on the S protein of other coronaviruses can also be shielded from immune recognition either via their “buried” location within the S or due to glycosylation [85,86,87,88]. However, dominant immunogenic epitopes are found on the S1 subunit, as this is the peripheral fragment of the enveloped S glycoprotein, and in the pre-fusion state, it is the main target of the immune response responsible for induction of high levels of antibodies [89]. In particular, the receptor-binding domain of IBV S may contain prime vaccine candidates, due to their ability to induce strong immune responses, as illustrated with other coronaviruses [35,90,91], and immunization with the entire RBD (19–237 aa) of IBV S might be an alternative vaccination. The HVR1 (38–67 aa) of IBV S contains a neutralising epitope at 24–61 aa [18,21,67,80]. We chose three epitope sequences for further examination based on multiple parameters, including surface accessibility, proximity to the HVR1, and location on the head of the S in the receptor-binding domain, as they may produce neutralising antibodies. Although some of the epitopes from the panel identified here coincided well with sites where neutralising antibodies are experimentally detected to bind, it still remains to be elucidated if they possess neutralising activity.

Towards the rational design of broad cross-protective vaccines against IBV with high efficacy and that can elicit strong neutralising responses in hosts, an appealing strategy would be to target a combination of epitope domains. This approach has been successful against the MERS-CoV, targeting epitopes which were non-RBD S1-specific, and preventing the emergence of escape mutants with amino acid substitutions in the RBD that was reported with the use of a single mAb [86]. One cautionary note is that targeting immunodominant non-neutralising epitopes on the receptor-binding domain may potentiate viral-mediated immune evasion mechanisms, as depicted with the HIV gp120 vaccine design [92,93,94]. Antibody binding to non-neutralising epitopes may block those able to bind to neutralising epitopes via sterical hindrance, as demonstrated with SARS-CoV, with S1-specific mAbs blocking the binding of neutralising antibodies to the S protein [95].

Glycans have an important role in post-translation modifications and protein folding, with N-linked glycosylation sites in the IBV S protein differentially affecting spike-membrane fusion, viral infectivity, and replication [96]. The receptor-binding domain of IBV S is heavily glycosylated with multiple N-glycosylation sites demonstrated to be essential for recognition of receptors and binding to host tissue, e.g., tracheal sections [66,97,98]. The panel of mAbs selected here to assess the specificity of the epitope arrays was initially raised against the IBV D207 virus [17,21]. Following the de-glycosylation of IBV S, the degree of antibody binding by these mAbs was reduced, indicating that N-glycan residues and protein conformation were important for antibody recognition [22]. The glycosylation of viral glycoproteins could be a mechanism of immune evasion via masking with host glycans, similar to what is evident with the HA of the Influenza A virus [99] and the S of MERS-CoV [80,85], SARS-CoV-1 [85,100], feline coronavirus [88], and SARS-CoV-2 [101,102]. The heterogeneity of the glycans presented on IBV M41 S was shown to exert influence upon viral replication; however, no direct link to alterations in neutralising activity was demonstrated [103]. The role of glycan moieties on the IBV S, whether directly immunogenic or shielding epitopes, has yet to be fully elucidated and warrants further investigation due to similarities with other coronaviruses.

Based on the known characteristics of IBV S and the epitopes identified here, three sequences were selected based on the following criteria: (1) spatially separated, (2) in the S1-NTD (likely to contain the RBD), and (3) with any proximity to the hypervariable regions (HVR1 is possibly associated with virus-neutralising activity). These three sequences were selected for further assessment of antibody recognition by synthetic peptide ELISAs. All selected epitopes were recognised to varying degrees by experimentally derived immune chicken serum from the rIBV vaccinated groups. Variability in host genetics may play a role in the generation of differential activities of species-specific antibodies to same B-cell epitopes [104]. As the outbred chicken line used here has heterogeneous histocompatibility (MHC) haplotypes [105], it is not surprising that there is a variation in the recognition of the epitopes. MHC B-locus haplotypes can influence susceptibility to IBV [106,107]. Inherent differences in the MHC B-locus of chickens can pre-determine the correlates of immune response and resistance to infection, with differences in IBV-specific antibody titres and IBV viral load seen across different lines [108]. Chickens with MHC haplotype B2 have significantly higher humoral responses, and there were no differences in innate responses, measured by IFNβ and IFNγ levels, compared to those with the MHC B19 haplotype [109].

## 5. Conclusions

In summary, the present study demonstrated the application of screening CLIPS arrays as a useful tool for mapping the antigenic regions of the S1 of IBV serotypes M41, QX, and 4/91, in an attempt to identify epitopes that might be associated with cross-protection. The screening of serum samples from rIBV vaccination experiments resulted in the detection of 52 highly immunogenic epitopes in the S1 sequences. These epitopes were assigned to six overlapping epitope binding regions and mapped onto the structural model of the IBV M41 S. A comparison with existing data on IBV S epitopes showed that some of the epitopes identified here coincided well with sites where neutralising antibodies are experimentally detected to bind. Notably, our study also validated that synthetic peptide mimics of the three epitope sequences selected for further investigation, due to their accessibility, were recognised, to a varying degree, by complex polyclonal serum. However, the amino acid residues responsible for controlling the structural conformation and properties of these epitopes are not yet determined. Therefore, for a true appreciation of the potential of these epitopes to contribute towards the rational design of broad-spectrum IBV vaccines, it is necessary to conduct site-directed mutagenesis to identify changes in residue binding affinity.

## Figures and Tables

**Table 1:** Type and number of peptides displayed on the IBV S1 CLIPS peptide arrays.

Type	CLIPS Description ^1^	Number of Peptides ^2^
Linear	All overlapping linear 15-mers (offset of one residue)	1574
Looped	Constrained linear 15-mers (flanked by Cys residues) on mP2 CLIPS	1574
Helical	Constrained 15-mers with additional Cys residues on mP2 CLIPS	1564
Coils	Structured 15-mers with additional Ile residues	1553

Nomenclature used for residues: Cys = Cysteine, Ile = Isoleucine. ^1^ All target sequences used had an offset of one residue. ^2^ Number of peptides displayed collectively across all three IBV S1 peptide arrays.

## Data Availability

Data are contained within the article and supplementary materials.

## Supplementary Materials

The following supporting information can be downloaded at: https://www.mdpi.com/article/10.3390/v15112279/s1. Figure S1: Alignment of IBV S1 gene sequences from M41, 4/91, and QX. Figure S2: Heat maps showing recognition of peptide sequences by pools of Mock, Mock/QX, and individual sera from homologous vaccinated/challenged chickens (14 dpc). Figure S3: Heat maps showing recognition of peptide sequences by binding to IBV S1 arrays by individual mapped sera from homologous vaccinated/challenged chickens at 14 dpc. Figure S4: Heat maps showing recognition of peptide sequences on IBV S1 arrays by individual mapped sera from heterologous vaccinated/challenged chickens at 14 dpc.
