# Supplementary material for: Revealing Novel-Strain-Specific and Shared Epitopes of Infectious Bronchitis Virus Spike Glycoprotein Using Chemical Linkage of Peptides onto Scaffolds Precision Epitope Mapping
Source: Viruses. 2023 Nov 20;15(11):2279. doi: 10.3390/v15112279 (PMC10675791; doi:10.3390/v15112279)
Supplement: Supplementary file 1 [file viruses-15-02279-s001.zip › viruses-2535707-supplementary.pdf]

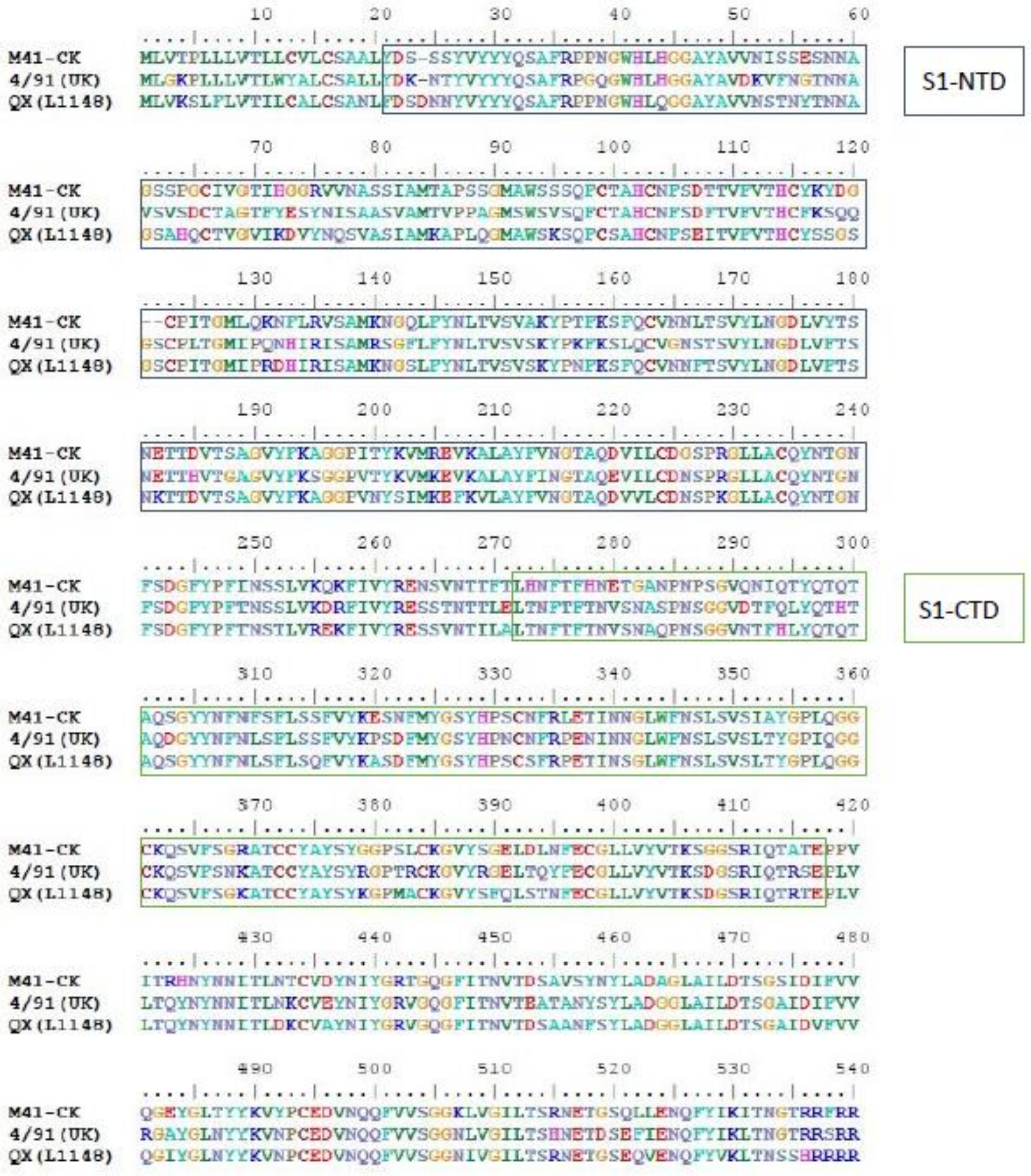

Supplementary Figure S1. Alignment of IBV S1 sequences from M41-CK, 4/91 and QX serotypes. The S1 subunitsfrom M41-CK (GenBank: MK728875.1), 4/91(UK) (GenBank: JN192154.1) and QX(L1148) (GenBank: KY933090.1) were aligned using ClustalW and the S1-NTD and S1-CTD, highlighted by boxesinblue and green respectively.The positions of the S1-NTD and S1-CTD areas determined by Shang et al 2018(https://doi.org/10.1371/journal.ppat.1007009).

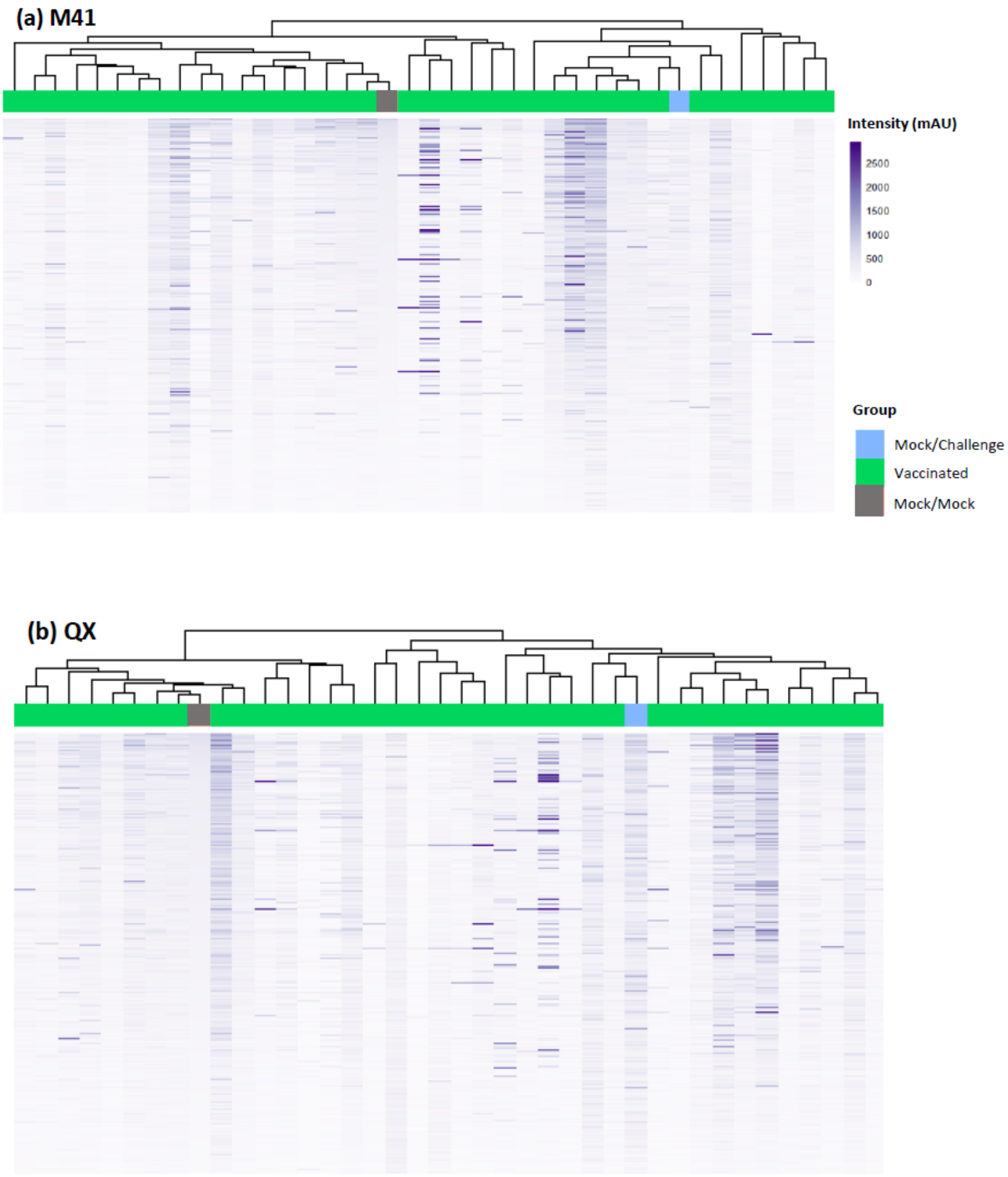

Supplementary Figure S2. Heat maps showing recognition of peptide sequences by pools of Mock/Mock, Mock/QX alongside individual sera from rIBV vaccinated homologous/vaccinated challenged chickens (collected at 14dpc).

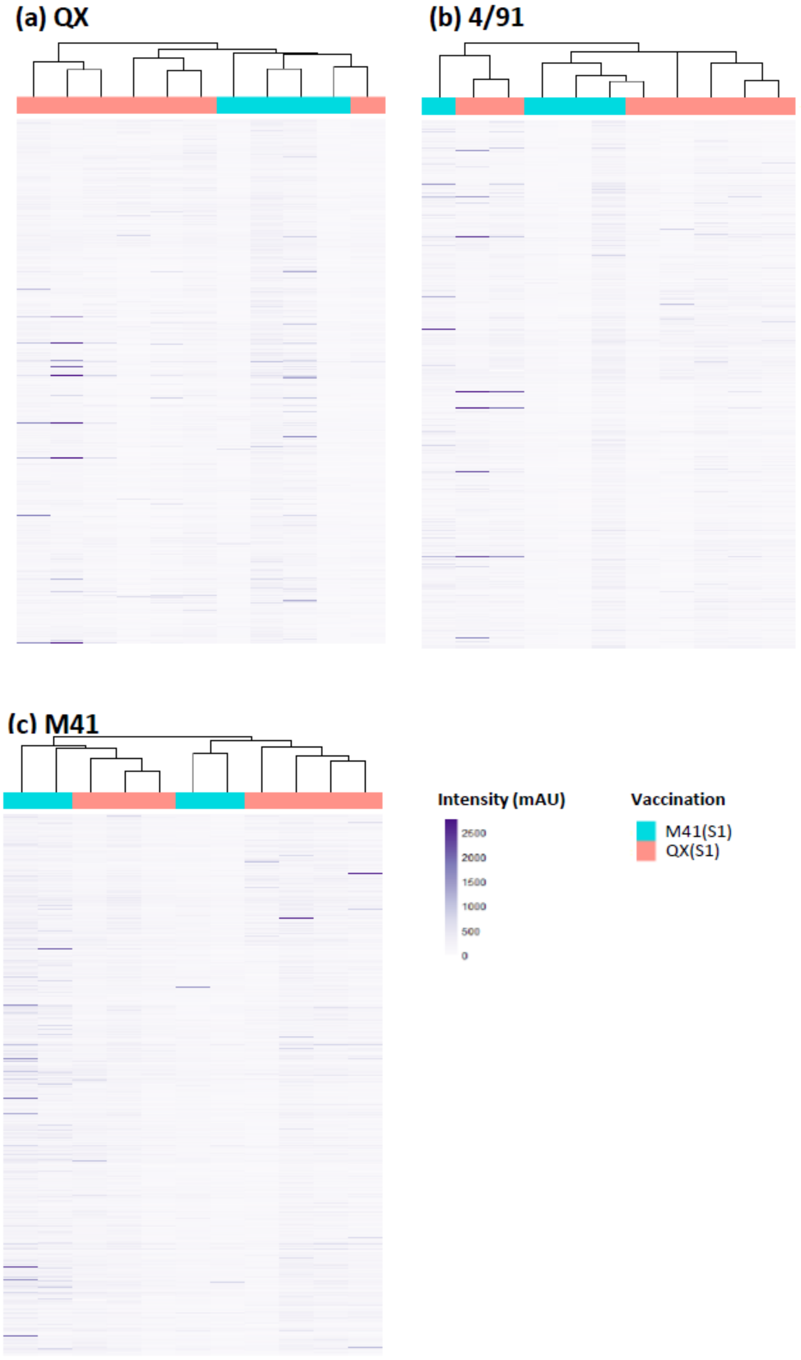

Supplementary Figure S3. Heat maps showing recognition of peptide sequences by individual mapped sera from homologous vaccinated/challenged chickens (at 14 dpc) by binding to IBV S1 arrays, QX, 4/91 and M41.

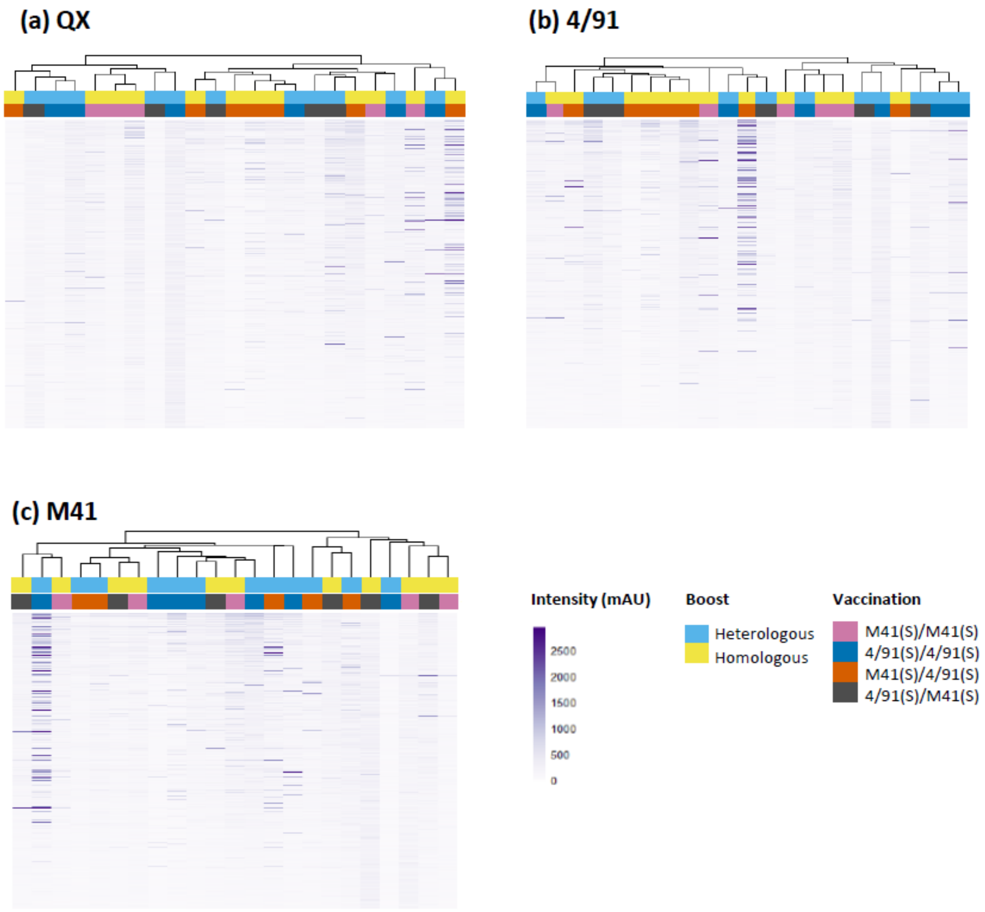

Supplementary Figure S4. Heat maps showing recognition of peptide sequences by individual mapped sera from heterologous vaccinated/challenged chickens (at 14 dpc) by binding to IBV S1 arrays, QX, 4/91 and M41.
